# Asymptotic Solution and Numerical Simulation of Lamb Waves in Functionally Graded Viscoelastic Film

**DOI:** 10.3390/ma12020268

**Published:** 2019-01-15

**Authors:** Xiaoshan Cao, Haining Jiang, Yan Ru, Junping Shi

**Affiliations:** School of Civil Engineering, Xi’an University of Technology, Xi’an 710048, China; 2160720004@stu.xaut.edu.cn (H.J.); ruyan@xaut.edu.cn (Y.R.); shijp@xaut.edu.cn (J.S.)

**Keywords:** Lamb wave, functionally graded viscoelastic material, minimum module approximation method, damping coefficient

## Abstract

To investigate Lamb waves in thin films made of functionally graded viscoelastic material, we deduce the governing equation with respect to the displacement component and solve these partial differential equations with complex variable coefficients based on a power series method. To solve the transcendental equations in the form of a series with complex coefficients, we propose and optimize the minimum module approximation (MMA) method. The power series solution agrees well with the exact analytical solution when the material varies along its thickness following the same exponential function. When material parameters vary with thickness with the same function, the effect of the gradient properties on the wave velocity is limited and that on the wave structure is obvious. The influence of the gradient parameter on the dispersion property and the damping coefficient are discussed. The results should provide nondestructive evaluation for viscoelastic material and the MMA method is suggested for obtaining numerical results of the asymptotic solution for attenuated waves, including waves in viscoelastic structures, piezoelectric semiconductor structures, and so on.

## 1. Introduction

Lamb waves, which are a type of plain strain wave in a thin film or a plate with a traction-free boundary, are widely used in nondestructive evaluation. Early research reported on Lamb waves focused on isotropic elastic plates [1]. Since then, scientists have directed more attention to Lamb waves in plates made of various materials, including viscoelastic materials [2], functionally graded materials (FGMs) [3], piezoelectric materials [4], and piezoelectric–piezomagnetic materials [5]. To detect material properties or damage to the structures, much research has been focused on guided waves in composite structures based on numerical and experimental methods [6,7]. 

FGMs were proposed by scientists as a kind of thermal-protection material in the 1990s [8]. In FGM structures, the material parameters are not constant and vary along one direction continuously. With the development of material technology, the FGM technique has been used not only for common elastic material but also for some smart materials, including piezoelectric [9,10] and piezoelectric–piezomagnetic materials [11]. To evaluate the mechanical properties of FGM structures, researchers have investigated various elastic waves in FGM structures, such as Lamb waves, horizontal shear (SH) waves [12], Love waves [13], and Rayleigh waves [14]. 

To address wave propagation problems in heterogeneous media, both numerical and analytical methods are employed for solving the wave-governing differential equations with variable coefficients. The main idea of numerical methods is to divide the functionally graded material into multilayer models and to simplify each sublayer as a homogenous layer [15,16,17,18]. Scientists have also proposed some analytical solutions for wave propagation problems in different heterogeneous structures. These methods include exact analytical expressions for material parameters following the same exponential function [19], the Wentzel–Kramers–Brillouin (WKB) method for large-wave-number [20] or cutoff problems [21,22], and the special function method for material parameters following some special function [23]. In recent decades, researchers suggested that these equations can be solved by using a power series method [11,24] and a Legendre polynomial method [25,26], which are fit for solving the wave propagation problem in heterogeneous structures in arbitrary cases in which material parameters vary continuously and slowly. The form of the dispersion equations based on these two methods contains series items. Therefore, these dispersion equations should be solved numerically.

It is found that not only elastic materials but also viscoelastic materials in nature have gradient properties. For example, when a material undergoes subsurface aging or subsurface damage, the elastic modulus varies along the thickness of the damaged subsurface region and mechanical gradient characteristics appear [27]. This should also occur for viscoelastic materials. For the wave propagation problem in viscoelastic structures, Lu et al. [28] found that the attenuation of Lamb waves increases with the increase of the thickness of the viscoelastic layer and that the mode is transformed as well. Compared with the propagation characteristics of Love waves in an elastic medium, the energy of Love waves in a Kelvin–Voigt viscoelastic medium is obviously attenuated, as shown by Zhang et al. [29]. SH waves have one displacement component. Yu et al. [30] deduced the dispersion equations for SH waves in orthotropic viscoelastic hollow cylinders. There are few studies on the propagation of Lamb waves with two displacement components in viscoelastic complex structures, and most of them use the Legendre polynomial method [31]. 

The dispersion equations for wave propagation in a viscoelastic material comprise a set of complex coefficient transcendental equations. To solve the transcendental equations with complex variables, Qian et al. [32] comprehensively analyzed the applicability of the parabolic Newton iteration method, the binary dichotomy method, and the modulus value convergence method. However, when the power series method is employed to solve the wave propagation problem in a functionally graded viscoelastic material (FGVM) structure, the dispersion equation, which is a transcendental equation with complex numbers in series form, is difficult to solve based on the above numerical simulation method. For example, the Newton iteration method requires that the solution be in the form of a display function rather than a series, while the binary dichotomy method and the modulus value convergence method might lead to the existence of spurious solutions.

In this study, we investigate the dispersion and attenuation characteristics of Lamb wave propagation in a thin film made of FGVM, which follows the Kelvin–Voigt model [33]. The governing equations with a displacement function are deduced and the power series asymptotic solution is obtained by using the power series method. Because the series has no explicit expression for the function, we propose the minimum module approximation (MMA) method for solving the complex coefficient dispersion equation. The detailed process of the MMA method, the existence analysis of its solution, and its optimization are given. The reliability of the power series solution is verified by comparison with the exact analytical solution for Lamb wave propagation in a functionally graded viscoelastic film. The dispersion and attenuation characteristics of Lamb wave propagation under different gradient parameters are discussed, and the damping coefficients are analyzed. Conclusions based on these results can provide a theoretical basis for nonhomogeneous viscoelastic structure nondestructive testing.

## 2. Basic Equation for Lamb Waves in FGVM Film

**Figure 1:**
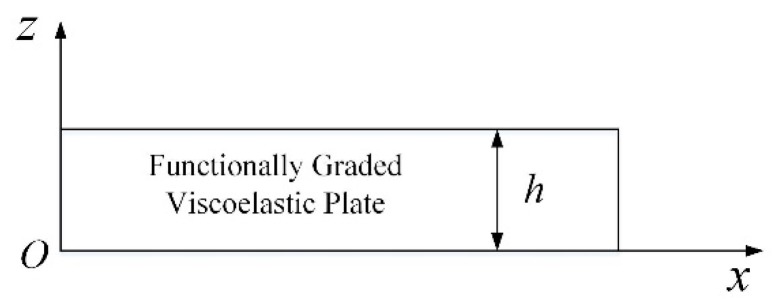
Functionally graded viscoelastic film and coordinate system.

Consider Lamb waves propagating in an isotropic functionally graded viscoelastic film along the *x* direction, as shown in Figure 1. The thickness of the film is *h*. The *z* direction is along the thickness direction. Let *u*, *v*, and *w* represent the displacement in the *x*, *y*, and *z* directions, respectively. For Lamb waves propagating is this structure, the displacement should satisfy:

(1) u=u(x,z,t),v=0, w=w(x,z,t)

If we let subscripts 1, 2, and 3 represent *x*, *y*, and *z*, respectively, then the stress tensor $\sigma_{ij}$, i,j=1−3, can be divided into two parts: the deviation stress ${\bar{\sigma }}_{ij}$ and the spherical stress tensor $\sigma_{m}$, where $\sigma_{m}=\sigma_{kk}/3$, k=1−3, and the repeated index in the subscript implies summation with respect to that index.

In the Kelvin model, the constitutive equation can be expressed as:

(2) $${\bar{\sigma }}_{ij}=2GS_{ij}+2\eta \frac{\partial S_{ij}}{\partial t},\;\sigma_{m}=\sigma_{kk}/3=KS_{kk}$$

where i,j,k,l=1−3, $S_{kl}$ is the strain tensor, *η* represents the viscosity coefficient, *G* is the shear modulus, and *K* is the bulk modulus. 

Equation (2) can be rewritten as:

(3) $$\sigma_{ij}=c_{ijkl}S_{kl}+{\bar{c}}_{ijkl}\frac{\partial S_{kl}}{\partial t}$$

where $c_{ijkl}$ and ${\bar{c}}_{ijkl}$ are components of the elastic tensor and viscosity tensor, respectively. In an FGVM, both these elastic parameters as well as the mass density *ρ* are not constants but are functions of *z*. 

The motion equations have the following form:

(4) $$\frac{\partial \sigma_{x}}{\partial x}+\frac{\partial \sigma_{xz}}{\partial z}=\rho \frac{\partial^{2}u}{\partial t^{2}},\;\frac{\partial \sigma_{xz}}{\partial x}+\frac{\partial \sigma_{z}}{\partial z}=\rho \frac{\partial^{2}w}{\partial t^{2}}$$

The relation between the strain and the displacement deduced from Equation (1) is:

(5) $$\varepsilon_{x}=\frac{\partial u}{\partial x},\;\varepsilon_{z}=\frac{\partial w}{\partial z},\;\gamma_{xz}=\frac{\partial u}{\partial z}+\frac{\partial w}{\partial x},\;\varepsilon_{y}=\gamma_{xy}=\gamma_{yz}=0$$

By using index reduction, the original fourth-order elastic parameters can be rewritten to second-order elastic parameters. By substituting Equation (5) into the constitutive Equation (3), we obtain the following component forms of the stress:

(6) $$\begin{array}{l} \sigma_{x}=c_{11}\frac{\partial u}{\partial x}+c_{13}\frac{\partial w}{\partial z}+{\bar{c}}_{11}\frac{\partial^{2}u}{\partial x\partial t}+{\bar{c}}_{13}\frac{\partial^{2}w}{\partial z\partial t}\\ \sigma_{xz}=c_{44}\left(\frac{\partial u}{\partial z}+\frac{\partial w}{\partial x}\right)+{\bar{c}}_{44}\left(\frac{\partial^{2}u}{\partial z\partial t}+\frac{\partial^{2}w}{\partial x\partial t}\right)\\ \sigma_{z}=c_{13}\frac{\partial u}{\partial x}+c_{11}\frac{\partial w}{\partial z}+{\bar{c}}_{13}\frac{\partial^{2}u}{\partial x\partial t}+{\bar{c}}_{11}\frac{\partial^{2}w}{\partial z\partial t} \end{array}$$

where the parameters in isotropic materials satisfy $c_{11}-c_{13}=2c_{44}$ and ${\bar{c}}_{11}-{\bar{c}}_{13}=2{\bar{c}}_{44}$.

Substitution of Equation (6) into Equation (4) leads to the following governing equations with respect to the displacement components:

(7) $$\begin{array}{c} \begin{array}{l} c_{11}\frac{\partial^{2}u}{\partial x^{2}}+c_{13}\frac{\partial^{2}w}{\partial x\partial z}+{\bar{c}}_{11}\frac{\partial^{3}u}{\partial x^{2}\partial t}+{\bar{c}}_{13}\frac{\partial^{3}w}{\partial x\partial z\partial t}+c_{44}\left(\frac{\partial^{2}u}{\partial z^{2}}+\frac{\partial^{2}w}{\partial x\partial z}\right)+{\bar{c}}_{44}\left(\frac{\partial^{3}u}{\partial z^{2}\partial t}+\frac{\partial^{3}w}{\partial x\partial z\partial t}\right)\\ +\frac{dc_{44}}{dz}\left(\frac{\partial u}{\partial z}+\frac{\partial w}{\partial x}\right)+\frac{d{\bar{c}}_{44}}{dz}\left(\frac{\partial^{2}u}{\partial z\partial t}+\frac{\partial^{2}w}{\partial x\partial t}\right)=\rho \frac{\partial^{2}u}{\partial t^{2}} \end{array}\\ \begin{array}{l} c_{44}\left(\frac{\partial^{2}u}{\partial x\partial z}+\frac{\partial^{2}w}{\partial x^{2}}\right)+{\bar{c}}_{44}\left(\frac{\partial^{3}u}{\partial x\partial z\partial t}+\frac{\partial^{3}w}{\partial x^{2}\partial t}\right)+c_{13}\frac{\partial^{2}u}{\partial x\partial z}+c_{11}\frac{\partial^{2}w}{\partial z^{2}}+{\bar{c}}_{13}\frac{\partial^{3}u}{\partial x\partial z\partial t}+{\bar{c}}_{11}\frac{\partial^{3}w}{\partial z^{2}\partial t}\\ +\frac{dc_{13}}{dz}\frac{\partial u}{\partial x}+\frac{dc_{11}}{dz}\frac{\partial w}{\partial z}+\frac{d{\bar{c}}_{13}}{dz}\frac{\partial^{2}u}{\partial x\partial t}+\frac{d{\bar{c}}_{11}}{dz}\frac{\partial^{2}w}{\partial z\partial t}=\rho \frac{\partial^{2}w}{\partial t^{2}} \end{array} \end{array}$$

Lamb waves propagating in a functionally graded viscoelastic film must satisfy not only the governing equations but also the traction-free conditions of the film, which are expressed as:

(8) $$\sigma_{z}\left(x,0\right)=0,\;\sigma_{xz}\left(x,0\right)=0,\;\sigma_{z}\left(x,h\right)=0,\;\sigma_{xz}\left(x,h\right)=0$$

## 3. Power Series Solution 

The solutions of the governing equations can be expressed as:

(9) u=U(z)exp[ik(x−ct)],w=−iW(z)exp[ik(x−ct)]

where i is the imaginary unit, *k* and *c* are the wave number and wave velocity, respectively, and *U*(*z*) and *W*(*z*) are the unknown amplitudes of the displacement. 

Substitution of Equation (9) into Equation (7) leads to:

(10) $$\begin{array}{l} {\hat{c}}_{44}U^{\prime \prime }+{\hat{c^{\prime }}}_{44}U^{\prime }+\left(\rho \Omega^{2}-{\hat{c}}_{11}k^{2}\right)U+k\left({\hat{c}}_{11}-{\hat{c}}_{44}\right)W^{\prime }+{{\hat{c}}^{\prime }}_{44}kW=0\\ {\hat{c}}_{11}W^{\prime \prime }+{\hat{c^{\prime }}}_{11}W^{\prime }+\left(\rho \Omega^{2}-{\hat{c}}_{44}k^{2}\right)W-k\left({\hat{c}}_{11}-{\hat{c}}_{44}\right)U^{\prime }-\left({{\hat{c}}^{\prime }}_{11}-2{{\hat{c}}^{\prime }}_{44}\right)kU=0 \end{array}$$

where ${\hat{c}}_{44}=c_{44}-i\Omega {\bar{c}}_{44}$, ${\hat{c}}_{11}=c_{11}-i\Omega {\bar{c}}_{11}$, Ω=ck is the frequency, and the prime symbol (′) represents differentiation with respect to thickness *z*. Both ${\hat{c}}_{44}$ and ${\hat{c}}_{11}$ are functions of *z* and Ω. Suppose that the material parameters vary along the thickness direction slowly, so that, for a certain Ω, the material parameters of the isotropic FGVM film can be expressed as follows:

(11) $${\hat{c}}_{11}=f_{1}\left(\frac{z}{h}\right),\;{\hat{c}}_{44}=f_{2}\left(\frac{z}{h}\right),\;\rho =f_{3}\left(\frac{z}{h}\right)$$

Suppose that the material parameters can be expressed in the power series form:

(12) $$f_{i}\left(\frac{z}{h}\right)=\sum_{n=0}^{\infty }a_{n}^{\langle i\rangle }{\left(\frac{z}{h}\right)}^{n}\;\left(i=1,2,3\right)$$

Therefore, the solutions of Equation (10) can also be expressed in a power series form as:

(13) $$U\left(z\right)=\sum_{n=0}^{\infty }s_{n}{\left(\frac{z}{h}\right)}^{n},\;W\left(z\right)=\sum_{n=0}^{\infty }t_{n}{\left(\frac{z}{h}\right)}^{n}$$

By substituting Equations (11)–(13) into Equation (10) and equating the coefficient of (*z*/*h*)*^n^* to zero, the following recursive equations can be obtained:

(14) $$\begin{array}{c} \begin{array}{l} \sum_{l=0}^{n}\left(l+2\right)\left(l+1\right)a_{n-l}^{\langle 2\rangle }s_{l+2}+\sum_{l=0}^{n}\left(n-l+1\right)\left(l+1\right)a_{n-l+1}^{\langle 2\rangle }s_{l+1}+{\left(kh\right)}^{2}\sum_{l=0}^{n}\left(a_{n-l}^{\langle 3\rangle }c^{2}-a_{n-l}^{\langle 1\rangle }\right)s_{l}\\ +\left(kh\right)\sum_{l=0}^{n}\left(l+1\right)\left(a_{n-l}^{\langle 1\rangle }-a_{n-l}^{\langle 2\rangle }\right)t_{l+1}+\left(kh\right)\sum_{l=0}^{n}\left(n-l+1\right)a_{n-l+1}^{\langle 2\rangle }t_{l}=0 \end{array}\\ \begin{array}{l} \sum_{l=0}^{n}\left(l+2\right)\left(l+1\right)a_{n-l}^{\langle 1\rangle }t_{l+2}+\sum_{l=0}^{n}\left(n-l+1\right)\left(l+1\right)a_{n-l+1}^{\langle 1\rangle }t_{l+1}+{\left(kh\right)}^{2}\sum_{l=0}^{n}\left(a_{n-l}^{\langle 3\rangle }c^{2}-a_{n-l}^{\langle 2\rangle }\right)t_{l}\\ -\left(kh\right)\sum_{l=0}^{n}\left(l+1\right)\left(a_{n-l}^{\langle 1\rangle }-a_{n-l}^{\langle 2\rangle }\right)s_{l+1}-\left(kh\right)\sum_{l=0}^{n}\left(n-l+1\right)\left(a_{n-l+1}^{\langle 1\rangle }-2a_{n-l+1}^{\langle 2\rangle }\right)s_{l}=0 \end{array} \end{array}$$

where *s*_0_, *s*_1_, *t*_0_, and *t*_1_ are undetermined coefficients. For l≥2, all of the *s*_l_ and *t*_l_ are linear functions of *s*_0_, *s*_1_, *t*_0_, and *t*_1_.

To simplify calculating the relation between $s_{l}$, $t_{l}$ and *s*_0_, *s*_1_, *t*_0_, *t*_1_, let:

(15) $$\left(s_{0j},s_{1j},t_{0j},t_{1j}\right)=I$$

where *j* = 1–4 and **I** is a 4 × 4 unit matrix. Therefore, the solution of Equation (10) can be rewritten as:

(16) $$U\left(z\right)=\sum_{j=1}^{4}C_{j}\sum_{n=0}^{\infty }s_{nj}{\left(\frac{z}{h}\right)}^{n},\;W\left(z\right)=\sum_{j=1}^{4}C_{j}\sum_{n=0}^{\infty }t_{nj}{\left(\frac{z}{h}\right)}^{n}$$

where the constants *C_j_* (*j* = 1–4) are to be determined. For *n* = 0 and 1, *s_nj_* and *t_nj_* are defined by expression (15); for other values of *n*, *s_nj_*, and *t_nj_* can be determined by solving Equation (14).

By substituting (16) into the boundary conditions, we then obtain the following linear algebraic equations for determining constants *C_j_* (*j* = 1–4):

(17) $$\begin{array}{c} -\left({\hat{c}}_{11}^{0}-2{\hat{c}}_{44}^{0}\right)khxC_{1}+{\hat{c}}_{11}^{0}C_{4}=0\\ {\hat{c}}_{44}^{0}C_{2}+kh{\hat{c}}_{44}^{0}C_{3}=0\\ \sum_{j=1}^{4}\left\{\sum_{n=0}^{\infty }\left[-kh\left({\hat{c}}_{11}^{h}-2{\hat{c}}_{44}^{h}\right)s_{nj}+{\hat{c}}_{11}^{h}\left(n+1\right)t_{\left(n+1\right)j}\right]\right\}C_{j}=0\\ \sum_{j=1}^{4}\left\{\sum_{n=0}^{\infty }\left[{\hat{c}}_{44}^{h}\left(n+1\right)s_{\left(n+1\right)j}+{\hat{c}}_{44}^{h}kht_{nj}\right]\right\}C_{j}=0 \end{array}$$

The sufficient and necessary condition for the existence of a nontrivial solution is that the determinant of the coefficient matrix must vanish. Therefore, for the dispersion relation for Lamb waves, there exists:

(18) $$\left|T_{ij}\right|=0$$

where

$$\begin{array}{c} T_{11}=-kh\left({\hat{c}}_{11}^{0}-2{\hat{c}}_{44}^{0}\right),\;T_{14}={\hat{c}}_{11}^{0},\;T_{22}=1,\;T_{23}=kh,\\ T_{3j}=\sum_{n=0}^{\infty }\left[-kh\left({\hat{c}}_{11}^{h}-2{\hat{c}}_{44}^{h}\right)s_{nj}+{\hat{c}}_{11}^{h}\left(n+1\right)t_{\left(n+1\right)j}\right],\;T_{4j}=\sum_{n=0}^{\infty }\left[\left(n+1\right)s_{\left(n+1\right)j}+kht_{nj}\right] \end{array}$$

where *j* = 1–4, and other items of *T_ij_* equal zero. The superscripts 0 and *h* represent the material parameters at the bottom and upper surfaces, respectively. 

Owing to the existence of the complex relation, Equation (18) is a complex coefficient transcendental equation. In this paper, we suppose that *k* and the wavelength *λ* are both real numbers. The wave velocity *c* contains both real and imaginary parts. The real part of the wave velocity represents the phase velocity, and the imaginary part is related to the attenuation characteristic of the wave.

## 4. MMA Method and Optimization

### 4.1. MMA Method

For solving an equation with a complex variable, we should obtain a solution for which both the real part and the imaginary part of the equation should be zero. Consider the complex equation:

(19) f(z)=0⇒f(x,y)=0,

where *x* and *y* are the real and imaginary parts, respectively, of the complex variable *z*, and *f*, which is a function of *z*, is also complex. We suppose z=a+ib is the solution of Equation (19). It should satisfy the conditions:

(20) Re[f(a,b)]=0 and Im[f(a,b)]=0,

where Re[f(x,y)] and Im[f(x,y)] represent the real and imaginary parts of the function f(x,y).

Let

(21) G(x,y)={Mod[f(x,y)]}2={Re[f(x,y)]}2+{Im[f(x,y)]}2,

where Mod[f(x,y)] is the module of the function f(x,y) and the square of the module is expressed by the function *G*(*x*, *y*). 

**Figure 2:**
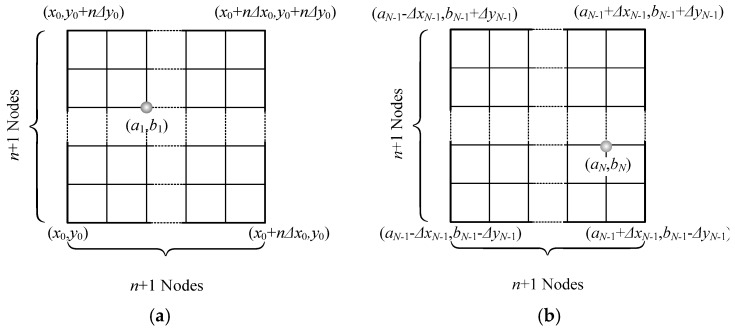
Steps of the MMA method. (**a**) First loop step, (**b**) *N*th loop step.

Suppose that the solution of Equation (19) exists and is unique in the region $\left(x_{0},{\bar{x}}_{0}\right)$, $\left(y_{0},{\bar{y}}_{0}\right)$. The model in Figure 2 is used to illustrate the solution steps. The first loop step is shown in Figure 2a: We divide the solution region into *n* × *n* grids, obtaining (*n* + 1)^2^ nodes in total, and calculate the module of each node by using Equation (21) and find the node (*a*_1,_ *b*_1_) satisfying:

(22) $$G\left(a_{1},b_{1}\right)=\min\left\{G\left(x_{0},y_{0}\right),G\left(x_{0}+i\Delta x_{0},y_{0}+j\Delta y_{0}\right)\right\}\;\left(i,j=1,\ldots ,n\right).$$

where

$$\Delta x_{0}=\frac{{\bar{x}}_{0}-x_{0}}{n},\;\Delta y_{0}=\frac{{\bar{y}}_{0}-y_{0}}{n}.$$

For the second loop step, based on *n*, we obtain the four points (*a*_1_−Δ*x*_0_,*b*_1_−*y*_0_), (*a*_1_+Δ*x*_0_,*b*_1_−*y*_0_), (*a*_1_+Δ*x*_0_,*b*_1_+*y*_0_), and (*a*_1_−Δ*x*_0_,*b*_1_+*y*_0_), remesh the square determined by the four points as the vertices into *n* × *n* grids once again, and also calculate the module of each node to find the minimum.

For the *N*th loop step (Figure 2b), we repeat the above step, obtaining:

(23) $$G\left(a_{N},b_{N}\right)=\min\left\{G\left(x_{N-1},y_{N-1}\right),G\left(x_{N-1}+i\Delta x_{N-1},y_{N-1}+j\Delta y_{N-1}\right)\right\}\;\left(i,j=1,\ldots ,n\right)$$

where $\left(a_{N},b_{N}\right)$ is the node with the minimum module after *N* time steps. The approximate solution of Equation (19) $z=a_{N}+ib_{N}$, can then be obtained.

### 4.2. Optimization of the MMA Method

The MMA method can be applied to solve equations with complex variables. To optimize the method, the following function *q* is introduced:

(24) $$q={\left(\frac{4}{n^{2}}\right)}^{\frac{1}{{\left(n+1\right)}^{2}}}$$

where *q* represents the average percentage of solution area reduction with each calculation and *n*^2^ and (*n* + 1)^2^ are the number of grids and nodes of the solution region, respectively.

To optimize the MMA method, we should find the *n* value needed to satisfy that *q* reaches its minimum. In other words, lnq should also be the minimum. Therefore, *n* should satisfy:

(25) $$\frac{d\ln q}{dn}=0$$

The solution of Equation (25) is *n* = 3.77. Because the number of grids needs to satisfy the condition of positive integers, we take the approximate solution at *n* = 4. 

In practical numerical analysis, we always predict with a certain level of uncertainly the region over which the equation with complex variables has a solution. If, in the first step, (*a*_1_, *b*_1_) lies on the boundary of the region, $\left(x_{0},{\bar{x}}_{0}\right)$, $\left(y_{0},{\bar{y}}_{0}\right)$, the solution might not lie in the region. In this case, *n* should be selected to be a larger number to certify the existence of the solution. However, a spurious solution might exist if the calculation region is too large. Normally, the solutions of these problems are always irrational. This means that we can find the solution as the module infinites approaches zero. We should check for the convergence of solution by testing the ratio of the module reduction in several continuous steps. For example, we can calculate the ratio of the module for every three loops and judge the convergence to avoid a spurious solution. 

It is worth noting that, if the minimum modulus is located at the boundary in the first calculation, there might be no solution in the computational domain. If the minimum modulus is not at the boundary after mesh refinement, the solution exists in the computational domain. Otherwise, the computational domain needs to be enlarged and recalculated. To verify the existence of the solution and avoid a spurious solution, we suggest that *n* should be selected as 6–8 in the first loop step in practical calculations.

## 5. Numerical Results and Discussion 

### 5.1. Comparison with the Exact Analytical Results 

To verify the validity of the power series method, the exact analytic solution and the asymptotic solution of the power series are compared when all material parameters vary with the same exponential function. The exact analytical solution can be obtained for waves propagating in the special FGVM thin film. The governing equations can be simplified to ordinary differential equations with constant coefficients, and the analytical solution can be obtained directly.

We suppose that the material parameters follow:

(26) $$\lambda =\lambda_{0}e^{p\left(z/h\right)},\;\mu =\mu_{0}e^{p\left(z/h\right)},\;\eta =\xi \mu =\xi \mu_{0}e^{p\left(z/h\right)},\;\rho =\rho_{0}e^{p\left(z/h\right)}$$

where $\lambda_{0},\;\mu_{0},\;\mathrm{and}\;\rho_{0}$ are material parameters of the film lower surface at z = 0, *η* is the viscosity coefficient, *ξ* is a constant and is selected as $10^{-5}$, and *p* represents the gradient parameter. The analytical solution in this condition can be selected as a reference for the solution of the power series reported in this paper.

The material parameters used in this paper can be deduced as:

(27) $$\begin{array}{c} {\hat{c}}_{11}=\left(\lambda_{0}+2\mu_{0}-\frac{4}{3}i\Omega \xi \mu_{0}\right)e^{p\left(z/h\right)}=\beta_{1}e^{p\left(z/h\right)}\\ {\hat{c}}_{44}=\left(\mu_{0}-i\Omega \xi \mu_{0}\right)e^{p\left(z/h\right)}=\beta_{2}e^{p\left(z/h\right)}\\ \rho =\rho_{0}e^{p\left(z/h\right)}=\beta_{3}e^{p\left(z/h\right)} \end{array}$$

The displacement amplitude can also be expressed in an exponential function form as:

(28) $$U_{e}\left(z\right)=Ae^{\alpha \left(z/h\right)}\;,\;W_{e}\left(z\right)=Be^{\alpha \left(z/h\right)}$$

By substitution of Equation (28) into Equation (10) the homogeneous linear equations for the undetermined coefficients *A* and *B* can be deduced as:

(29) $$\begin{array}{l} \left[\beta_{2}\alpha^{2}+p\alpha \beta_{1}+\left(\beta_{3}c^{2}-\beta_{1}\right){\left(kh\right)}^{2}\right]A+\left[kh\left(\beta_{1}-\beta_{2}\right)\alpha +khp\beta_{2}\right]B=0\\ \left[kh\left(\beta_{2}-\beta_{1}\right)\alpha +p\left(2\beta_{2}-\beta_{1}\right)\right]A+\left[\beta_{1}\alpha^{2}+p\alpha \beta_{1}+\left(\beta_{3}c^{2}-\beta_{2}\right){\left(kh\right)}^{2}\right]B=0 \end{array}$$

Equations (29) comprise a set of linear homogeneous equations with respect to *A* and *B*. From the necessary and sufficient conditions for the existence of a nontrivial solution, we obtain that the determinant of the coefficient matrix is equal to zero:

(30) $$\left|\begin{array}{cc} \beta_{2}\alpha^{2}+p\alpha \beta_{1}+\left(\beta_{3}c^{2}-\beta_{1}\right){\left(kh\right)}^{2} & kh\left(\beta_{1}-\beta_{2}\right)\alpha +khp\beta_{2}\\ kh\left(\beta_{2}-\beta_{1}\right)\alpha +p\left(2\beta_{2}-\beta_{1}\right) & \beta_{1}\alpha^{2}+p\alpha \beta_{1}+\left(\beta_{3}c^{2}-\beta_{2}\right){\left(kh\right)}^{2} \end{array}\right|=0$$

Considering that Equation (30) is a fourth-order equation, we suppose that the solution is $\alpha_{j}$ (*j* =1–4). The relation between *A* and *B* is derived by calculating Equation (29) as follows:

(31) $$B_{i}=f_{i}A_{i}\left(i=1–4\right)$$

The displacement amplitude solution of Equation (10) can be rewritten as:

(32) $$U=\sum_{j=1}^{4}A_{j}e^{\alpha_{j}\left(z/h\right)}\;W=\sum_{j=1}^{4}f_{j}A_{j}e^{\alpha_{j}\left(z/h\right)}$$

Similarly, by considering the boundary conditions, we then obtain the dispersion equation:

(33) $$\left|T_{ij}\right|=0$$

where

$$\begin{array}{c} T_{1j}=\left(\beta_{1}-2\beta_{2}\right)k+\beta_{1}f_{j}\alpha_{j},\;T_{2j}=\alpha_{j}-kf_{j}\\ T_{3j}=\left[\left(\beta_{1}-2\beta_{2}\right)k+\beta_{1}f_{j}\alpha_{j}\right]e^{\alpha_{j}h},\;T_{4j}=\left(\alpha_{j}-kf_{j}\right)e^{\alpha_{j}h}\left(j=1-4\right) \end{array}$$

In numerical analysis, the normalized wave velocity $\hat{c}$ and the dimensionless wave number *kh* are applied for describing the wave propagation property. The normalized dimensionless wave velocity $\hat{c}$ satisfies:

(34) $$\hat{c}=c/c_{sh}$$

where $c_{sh}=\sqrt{G/\rho }$, which is the bulk shear wave velocity. The Poisson ratio is a constant and satisfies ν = 0.25. 

**Figure 3:**
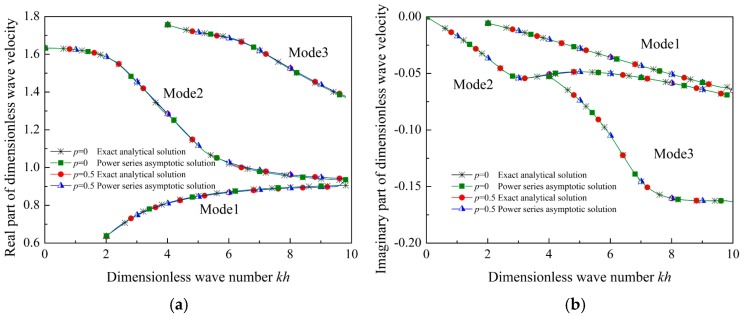
Wave velocity of Lamb waves in homogenous viscoelastic film and in special FGVM film. (**a**) Real part, (**b**) imaginary part.

To evaluate the accuracy and precision of the power series and MMA methods, the relation between the normalized wave velocity and the dimensionless wave number for Lamb waves propagating in the special FGVM thin film is plotted in Figure 3. When *p* = 0, the FGVM thin film becomes a homogenous viscoelastic thin film. Figure 3a,b present the real and imaginary parts of the normalized wave velocity, respectively. It is found that the solution obtained by using the power series method agrees well with the exact analytical solution. 

By checking the results of the change of phase velocity, we find that there exists little difference between the wave velocity curves for Lamb waves in the homogenous viscoelastic thin film and in the special FGVM thin film. Normally, the dispersion curves of Lamb waves in homogenous film can be determined by bulk shear wave velocity and the Poisson ratio. Both the bulk shear wave velocity and the Poisson ratio in homogenous thin film are same as those in the special FGVM thin film. It can be used to explain that the dispersion curves of the two cases are almost identical. This implies that we cannot measure the gradient parameters by variation of the wave velocity

We further study the wave structure of Lamb wave propagation in different viscoelastic thin films. The normalized displacement amplitude is defined as:

(35) ℏu=|U|/|U(0)|⋅sign{Re(U)/Re[U(0)]}, ℏw=|W|/|U(0)|⋅sign{Re(W)/Re[U(0)]}

where U(0), which represents displacement component at z = 0, is selected to be 1 in the numerical analysis, Re is the real part of the complex number, and the sign function satisfies

(36) $$\mathrm{sign}(x)=\{\begin{array}{cc} 1, & x\geq 0\\ -1, & x<0 \end{array}$$

**Figure 4:**
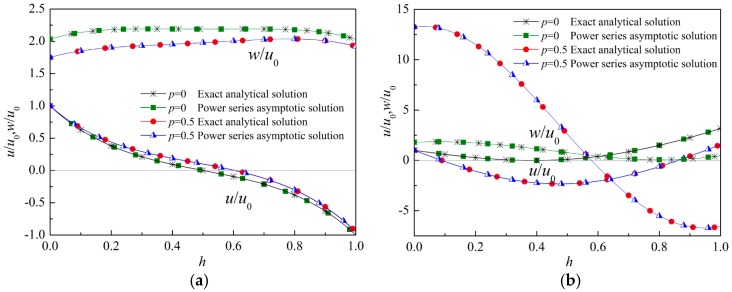

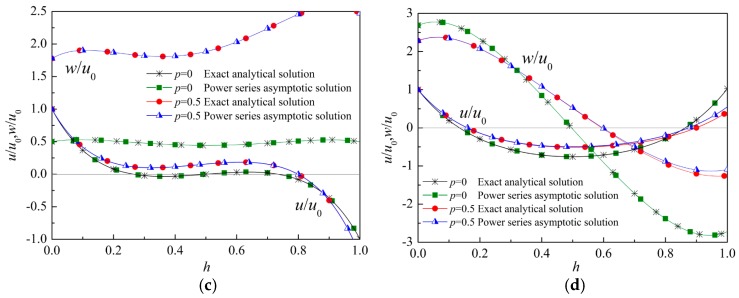
Wave structure. (**a**) kh=π, Mode 1, (**b**) kh=π, Mode 2, (**c**) kh=2π, Mode 1, (**d**) kh=2π, Mode 2.

The normalized displacement amplitude of the first two modes at kh=π and kh=2π are plotted in Figure 4. The curves obtained from the exact solution and these obtained by using the power series method coincide completely. In a homogenous viscoelastic thin film, the wave structure is symmetric or antisymmetric. However, because of the asymmetric properties of the FGVM thin film, the displacement amplitudes are not symmetric. 

**Figure 5:**
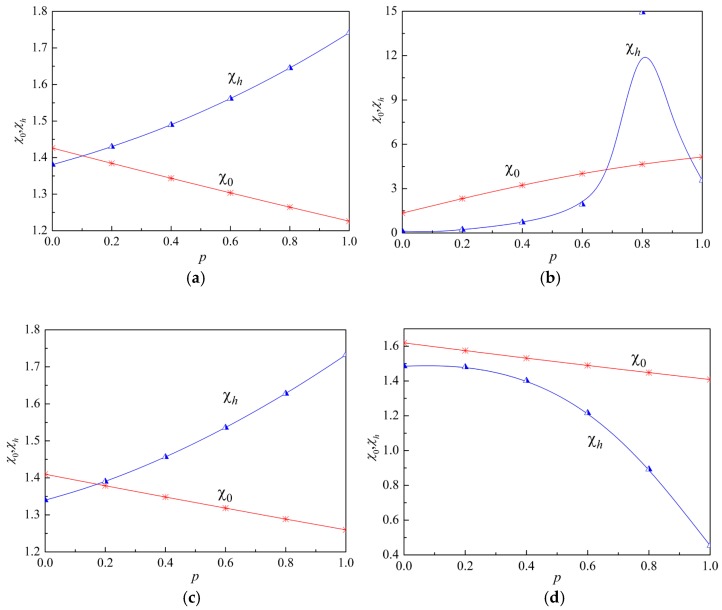
Ellipticity of particles on lower and upper surfaces versus the gradient parameter. (**a**) kh=π, Mode 1, (**b**) kh=π, Mode 2, (**c**) kh=2π, Mode 1, (**d**) kh=2π, Mode 2.

To further investigate the influence of the gradient property on the displacement, we denote the ellipticity of particles on lower and upper surfaces as $\chi_{0}=\left|w_{0}/u_{0}\right|$ and $\chi_{h}=\left|w_{h}/u_{h}\right|$, respectively. The relation between the gradient parameter and the ellipticity of particles at kh=π and kh=2π is plotted in Figure 5. It is found that the influence of the gradient property on the ellipticity of a particle on the surface is more obvious than that on the wave velocity. 

### 5.2. Material Parameters Varying Linearly

For numerical analysis with the theoretical model described above, we assumed that the Lamé parameters λ and μ, mass density ρ, and viscosity coefficient η in the functionally graded viscoelastic film varied as follows:

(37) $$\lambda =\lambda_{0}+p_{1}\lambda_{0}\frac{z}{h},\;\mu =\mu_{0}+p_{2}\mu_{0}\frac{z}{h},\;\rho =\rho_{0}+p_{3}\rho_{0}\frac{z}{h},\;\eta =\xi \mu$$

where $\lambda_{0},\;\mu_{0},\;\mathrm{and}\;\rho_{0}$ are material parameters of the film lower surface at z = 0; *η* is the viscosity coefficient; *ξ* is a constant and is selected as $10^{-5}$ (except in Section 5.2.3); and $p_{1},\;p_{2}\;\mathrm{and}\;p_{3}$ are the gradient parameters of λ, μ, and ρ, respectively $\left(0\leq p_{1},p_{2},p_{3}<1\right)$. 

The material parameters used in this paper can be deduced as:

(38) $${\hat{c}}_{11}=\lambda +2\mu -\frac{4}{3}i\Omega \eta ,\;{\hat{c}}_{44}=\mu -i\Omega \eta$$

#### 5.2.1. All Material Parameters Varying Identically 

**Figure 6:**
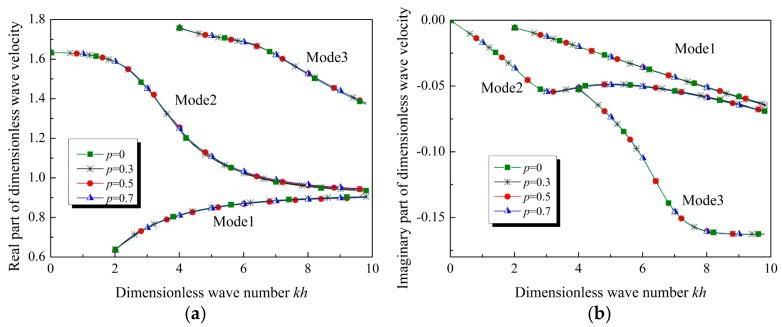
Wave velocity of Lamb waves in homogenous viscoelastic film and in FGVM film (with all material parameters varying linearly and identically). (**a**) Real part, (**b**) imaginary part.

Suppose that all material parameters vary along the thickness direction linearly and identically, i.e., $p_{i}=p\left(i=1,2,3\right)$. The wave velocity plotted as a function of wave number when *p* = 0, 0.3, 0.5, and 0.7 is shown in Figure 6. By comparing the results for the case in Section 5.1, a similar conclusion can be reached. If all the material parameters vary along the thickness direction identically, the wave velocity curves almost coincide. 

In these cases, the bulk wave velocity, including the shear wave velocity $c_{sh}$ and the longitudinal wave velocity $c_{L}$, where $c_{L}=\sqrt{\left(\lambda +2\mu \right)/\rho }$, are constants. This implies that, if the bulk wave velocities are constants, the wave velocity of Lamb waves in the FGVM thin film are almost similar to that in a homogenous film.

**Figure 7:**
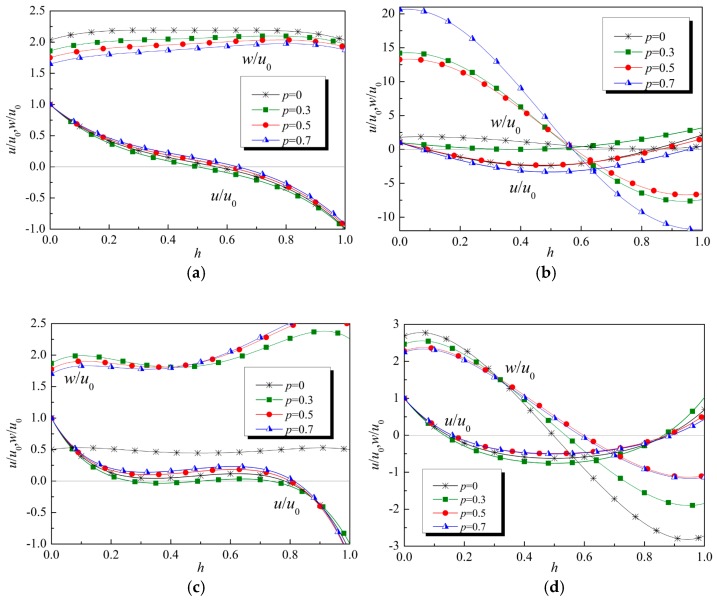
Wave structure of Lamb waves (with all material parameters varying linearly and identically). (**a**) kh=π, Mode 1, (**b**) kh=π, Mode 2, (**c**) kh=2π, Mode 1, (**d**) kh=2π, Mode 2.

Similarly, the wave structures are also plotted in Figure 7. It is found that the gradient parameter has an obvious influence on the wave structure. It is also shown in Figure 7 that the ellipticity of particles on the upper and lower surfaces is different owing to the gradient property of the FGVM film. Considering testability, we suggest that the ellipticity of a particle on the surface can be applied for measuring the gradient parameter when all material parameters vary identically. 

#### 5.2.2. Material Parameters Varying Independently 

To investigate the influence of the elastic modulus and density gradient on dispersion and attenuation characteristics of Lamb waves in gradient viscoelastic film, we chose three types of films for which the gradient parameters are:

$$A:\;p_{1}=p_{2}=p_{3}=0;\;B:\;p_{1},p_{2}=0,\;p_{3}=0.2;\;C:\;p_{1}=0.2,\;p_{2}=0.2,\;p_{3}=0$$

Film A is a homogeneous film, which can be used for referencing the propagation characteristics in the gradient film. The elastic modulus in film B is a constant, and the density increases along the thickness of the film and the density of the lower surface is the same as that of the homogeneous film. Conversely, the density in film C is a constant, and the elastic modulus varies along the thickness direction linearly.

**Figure 8:**
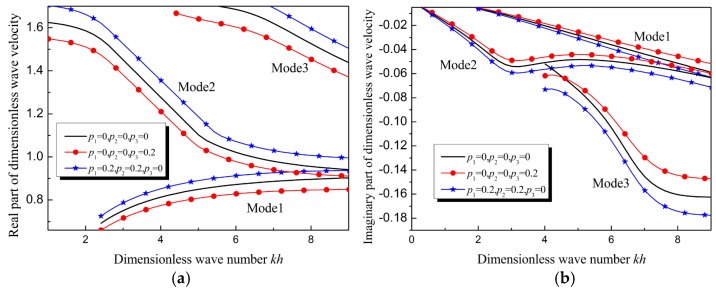
Dimensionless Lamb wave velocity in functionally graded viscoelastic films of different gradient parameters: (**a**) Dispersion curves of Lamb waves in the three films, (**b**) attenuation curves of Lamb waves in the three films.

The real and imaginary parts of the wave velocity in the three types of films are shown in Figure 8. When the mass density increases, the real part and the absolute value of the imaginary part of the wave velocity of each mode are less than these in the homogenous film; when the elastic modulus increases, the real part and the absolute value of the imaginary part of the dimensionless wave velocity of each mode are larger than these in the homogenous film.

#### 5.2.3. Relative Viscosity Coefficient Varying Independently 

In engineering application, the relative viscosity coefficient might vary along one direction because of the environment. However, in these cases, the mass density and the elastic parameters might not change. To reveal the influence of the gradient relative viscosity coefficient on the wave property, we suppose that the relative viscosity coefficient *ξ* varies along the thickness direction and that other parameters including λ, μ, and ρ are constants. The material parameters are:

(39) $$\lambda =\lambda_{0},\;\mu =\mu_{0},\;\rho =\rho_{0},\;\eta =\xi \mu ,\;\xi =10^{-5}p_{4}\left(z/h\right)$$

**Figure 9:**
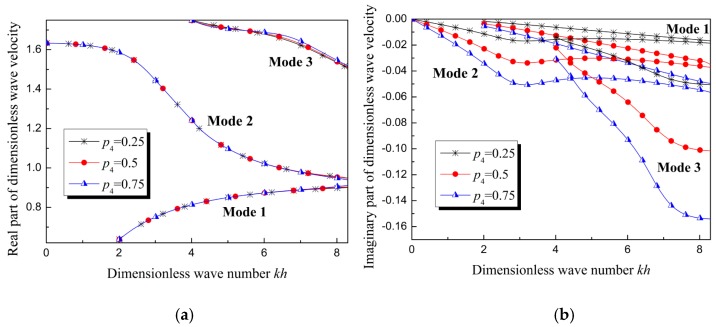
Wave velocity of Lamb waves in homogenous viscoelastic film and in FGVM film (with only the relative viscosity coefficient varying linearly and identically). (**a**) Real part, (**b**) imaginary part.

The wave velocity is plotted as function of wave number in Figure 9. In Figure 9a, the curves for the real part of the phase velocity almost coincide. This suggests that the influence of the gradient relative viscosity coefficient on the dispersion curves is too slight to measure. However, obvious differences can be observed in Figure 9b, which describes the relation between the imaginary parts of the wave velocity. The absolute value of the imaginary part of the dimensionless wave velocity increases with the increase of the gradient relative viscosity coefficient. The physical meaning of the imaginary parts of the wave velocity will be discussed in the next section. 

### 5.3. Influence of Gradient Parameter on Wave Attenuation 

Equation (18) is a complex equation. As the wave number is a real number, the wavelength is also a real number, the obtained wave velocity is complex, and the product *Ω* of the wave velocity *c* and the wave number *k* is complex, which can be expressed as follows:

(40) cp=Re(c),Ω=ω+iω˜=ck

where ω and $\tilde{\omega }$ are the real and imaginary parts of *Ω*, respectively, ω is frequency, and $\tilde{\omega }$ is related to the attenuation of the wave amplitude. From Equation (40), we have:

(41) $$\omega =c_{p}k$$

In viscoelastic materials, the wave propagation process is essentially a quasi-periodic motion, and the period of the particle displacement is determined by the phase velocity. The period is expressed as follows:

(42) T=2πkRe(c)=2πω

To analyze the attenuation trend, we define the amplitude ratio of the adjacent period as the damping coefficient *γ*, given by:

(43) γ=exp[−2πIm(c)Re(c)]=exp(−2πω˜ω)

In this study, the normalized product of frequency and thickness $\hat{\omega }h$ is selected to be the abscissa. If

$$\hat{\omega }=\frac{\omega }{c_{sh}}$$

is satisfied, then

(44) $$\hat{\omega }h=\frac{\omega }{c_{sh}}h=\frac{c_{p}}{c_{sh}}kh$$

**Figure 10:**
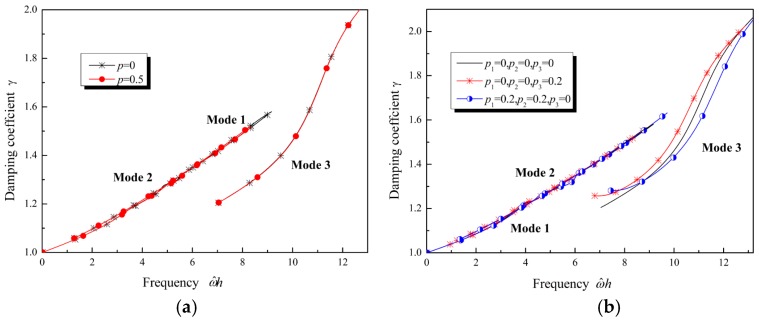
Damping coefficient of Lamb waves with Lamé parameters and density varying along the thickness direction: (**a**) Material parameters varying following the same exponential function; (**b**) material parameters varying following a linear function.

**Figure 11:**
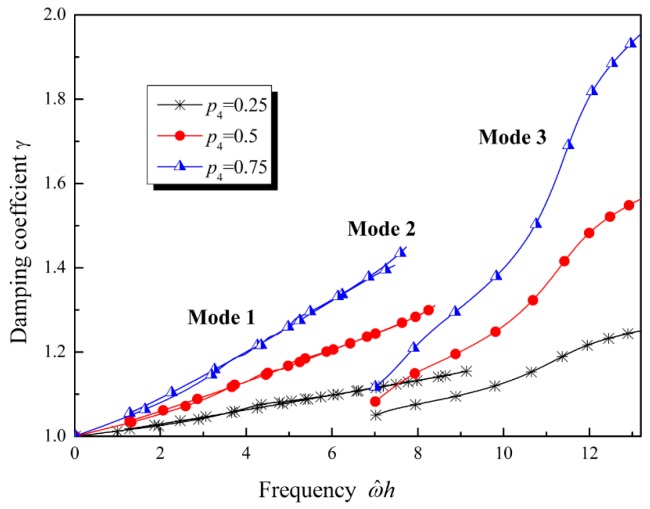
Damping coefficient of Lamb waves with the relative viscosity coefficient varying.

The influence of the gradient properties on the damping coefficient is plotted in Figure 10. The damping coefficient increases with the increase of the frequency. When material parameters are constants, or material parameters vary along the thickness direction with the same exponential function, or both Lamé parameters and mass density vary linearly, the damping coefficient of Mode 1 and Mode 2 is similar, as shown in Figure 10a,b. However, when the Lamé parameters and mass density do not vary identically, a difference in the damping coefficient can be observed, as shown in Figure 10b. When the mass density increases, the damping coefficient increases at high frequency ($\hat{\omega }h>s0$). This implies that, at high frequency, if the mass density increases along the thickness direction, then the Lamb waves in the FGVM thin film should attenuate more quickly than those in a homogenous material. Conversely, if the Lamé parameters increase along the thickness direction, then the attenuation of Lamb waves in the FGVM thin film should be weakened. If only the relative viscosity coefficient increases along the thickness direction, then the attenuation of Lamb waves will become more serious, as shown in Figure 11. As the gradient coefficient increases, the damping coefficient increases and the attenuation tendency becomes obvious. 

## 6. Conclusions

The power series method can be employed for solving the governing differential equations for Lamb wave propagation in FGVM thin films. The MMA method is proposed to solve the transcendental equations in the form of a series with complex coefficients. It is suggested that the meshing number should be selected as 6–8 in the first loop step and 4 in other loops. The numerical results obtained by these methods agree well with the exact analytical solution. 

When Lamé parameters and mass density vary along the thickness direction identically, the influence of the gradient properties on the wave velocity is slight but that on the wave structure and the ellipticity of particles on the surface is obvious. This suggests that the ellipticity of particles on the surface should be selected to measure the gradient property if the bulk wave velocities are constants in the FGVM thin film. When Lamé parameters and mass density vary along the thickness direction independently, the variation of the phase velocity can be used for testing the gradient parameters. However, when the relative viscosity coefficient is a variable and both Lamé parameters and mass density are constants, the gradient property will not affect the phase velocity. The attenuation tendency becomes obvious with the increase of the gradient relative viscosity coefficient.

The method proposed herein and the results obtained should provide theoretical guidance for ultrasonic nondestructive testing of heterogeneous viscoelastic materials and enable the safe evaluation of surface acoustic wave devices.
